# A Predicted Mannoprotein Participates in *Cryptococcus gattii* Capsular Structure

**DOI:** 10.1128/mSphere.00023-18

**Published:** 2018-04-25

**Authors:** Julia Catarina Vieira Reuwsaat, Heryk Motta, Ane Wichine Acosta Garcia, Carolina Bettker Vasconcelos, Bárbara Machado Marques, Natália Kronbauer Oliveira, Jéssica Rodrigues, Patrícia Aline Gröhns Ferrareze, Susana Frases, William Lopes, Vanessa Abreu Barcellos, Eamim Daidrê Squizani, Jorge André Horta, Augusto Schrank, Marcio Lourenço Rodrigues, Charley Christian Staats, Marilene Henning Vainstein, Lívia Kmetzsch

**Affiliations:** a Laboratório de Fungos de Importância Médica e Biotecnológica, Centro de Biotecnologia, Universidade Federal do Rio Grande do Sul, Porto Alegre, Brazil; b Laboratório de Biologia Celular de Leveduras Patogênicas, Instituto de Microbiologia Paulo de Goés, Universidade Federal do Rio de Janeiro, Rio de Janeiro, Brazil; c Laboratório de Ultraestrutura Celular Hertha Meyer, Instituto de Biofísica Carlos Chagas Filho, Universidade Federal do Rio de Janeiro, Rio de Janeiro, Brazil; d Departamento de Biologia e Farmácia, Universidade de Santa Cruz do Sul (UNISC), Programa de Pós-Graduação em Promoção da Saúde, Santa Cruz do Sul, RS, Brazil; e Centro de Desenvolvimento Tecnológico em Saúde, Fundação Oswaldo Cruz, Fiocruz, Rio de Janeiro, Brazil; f Departamento de Biologia Molecular e Biotecnologia, Universidade Federal do Rio Grande do Sul, Porto Alegre, Brazil; Yonsei University

**Keywords:** *Cryptococcus gattii*, capsular polysaccharide, mannoprotein

## Abstract

The yeast-like pathogen Cryptococcus gattii is an etiological agent of cryptococcosis. The major cryptococcal virulence factor is the polysaccharide capsule, which is composed of glucuronoxylomannan (GXM), galactoxylomannan (GalXM), and mannoproteins (MPs). The GXM and GalXM polysaccharides have been extensively characterized; however, there is little information about the role of mannoproteins in capsule assembly and their participation in yeast pathogenicity. The present study characterized the function of a predicted mannoprotein from C. gattii, designated Krp1. Loss-of-function and gain-of-function mutants were generated, and phenotypes associated with the capsular architecture were evaluated. The null mutant cells were more sensitive to a cell wall stressor that disrupts beta-glucan synthesis. Also, these cells displayed increased GXM release to the culture supernatant than the wild-type strain did. The loss of Krp1 influenced cell-associated cryptococcal polysaccharide thickness and phagocytosis by J774.A1 macrophages in the early hours of interaction, but no difference in virulence in a murine model of cryptococcosis was observed. In addition, recombinant Krp1 was antigenic and differentially recognized by serum from an individual with cryptococcosis, but not with serum from an individual with candidiasis. Taken together, these results indicate that C. gattii Krp1 is important for the cell wall structure, thereby influencing capsule assembly, but is not essential for virulence *in vivo*.

**IMPORTANCE** Cryptococcus gattii has the ability to escape from the host’s immune system through poorly understood mechanisms and can lead to the death of healthy individuals. The role of mannoproteins in C. gattii pathogenicity is not completely understood. The present work characterized a protein, Kpr1, that is essential for the maintenance of C. gattii main virulence factor, the polysaccharide capsule. Our data contribute to the understanding of the role of Kpr1 in capsule structuring, mainly by modulating the distribution of glucans in C. gattii cell wall.

## Abstract (precis)

Cryptococcus gattii has the ability to escape from the host’s immune system through poorly understood mechanisms and can lead to the death of healthy individuals. The role of mannoproteins in C. gattii pathogenicity is not completely understood. The present work characterized a protein, Kpr1, that is essential for the maintenance of C. gattii main virulence factor, the polysaccharide capsule. Our data contribute to the understanding of the role of Kpr1 in capsule structuring, mainly by modulating the distribution of glucans in C. gattii cell wall.

## INTRODUCTION

The sibling species Cryptococcus gattii and Cryptococcus neoformans are the main cause of cryptococcosis in animals and humans (1), a life-threatening disease with an annual incidence of nearly 280,000 cases (2). Globally, cryptococcal meningitis accounts for 15% of AIDS-related deaths and, if not properly threated, can cause up to 70% of the deaths of cryptococcosis patients (2). While C. gattii is mostly responsible for the infections of immunocompetent patients, C. neoformans has higher infection incidence in immunocompromised hosts (3). C. gattii infections were assumed to be restricted to tropical and subtropical areas, but the outbreak in 1999 on Vancouver Island, Canada, altered this view, confirming the presence of this species in temperate regions (4, 5). C. gattii is widespread in trees and soil, initiating human infection by the inhalation of spores or dried yeasts, which when they reach the lungs can spread through the bloodstream to the brain, causing meningitis (6).

During the host-pathogen interaction, *Cryptococcus* species use a repertoire of virulence strategies to survive and proliferate, including the production of melanin, secretion of enzymes such as phospholipase B and urease, as well as the production of a polysaccharide capsule that interacts with the cell wall (7). The capsule is considered the major cryptococcal virulence factor due to its immunosuppressive properties (8–12). It is composed of the polysaccharides glucuronoxylomannan (GXM) (90 to 95%) and galactoxylomannan (GalXM) (5 to 10%), with less than 1% of mannoproteins (MPs) (13, 14). Chitooligomers (15) and glucans (16), as well as some cytoplasmic proteins (heat shock proteins) (17), have also been identified as transitory components of the capsule, revealing the dynamics of this structure. C. neoformans GXM is the most characterized capsule component (18), and its functional characteristics are usually similar to those of C. gattii GXM (19). However, different GXM fractions produced by C. gattii and C. neoformans could develop distinct innate immune responses in host cells (20, 21), suggesting that C. gattii GXM is different from C. neoformans to some extent. It is also known that C. neoformans strains with mutations in gene products involved in galactose metabolism do not secret GalXM and are easily eradicated from the host, revealing the importance of this polysaccharide in the yeast pathogenicity (22).

The roles of mannoproteins in capsule structure and assembly are still not clear. However, their influence in the induction of T cell responses in the host is well established (23–25), indicating their potential as targets to immunotherapy. Mannoproteins are found in a large variety of fungi (26, 27), and their functions are closely related to cell wall structure (28, 29). Studies have demonstrated the function of mannoproteins in cryptococcal physiology or virulence. C. neoformans Cig1 is a mannoprotein that functions as a cell surface hemophore, and cells lacking *CIG1* displayed impaired growth in iron-limiting medium and a small capsule phenotype (30, 31). Moreover, the MP98 protein from C. neoformans was characterized as a chitin deacetylase that contributes to the maintenance of cell wall integrity (32). Despite their low abundance in the capsule, the role of mannoproteins in capsular architecture has never been established. This study determined that a C. gattii mannoprotein (Krp1) affects capsule assembly in the cell wall but does not influence yeast pathogenicity *in vivo*.

## RESULTS

### The predicted mannoprotein repertoire of C. gattii.

In order to characterize the set of predicted mannoproteins encoded by C. gattii R265, an *in silico* approach was used to identify proteins with mannoprotein signatures, specifically, the presence of a signal peptide, a glycosylphosphatidylinositol (GPI) anchor, and serine-threonine-rich sites that would comprise the glycosylation site (33). Employing a combination of SignalP (34), PredGPI (35), and GlycoEP (36) analyses, the presence of 34 mannoprotein-coding genes in C. gattii R265 genome was predicted (see Table S1 in the supplemental material). Profile assignments employing InterProScan (37) led to the identification of diverse conserved domains in 20 of these predicted proteins, most of them related to enzymes acting on carbohydrates (Table S1). Among such mannoprotein-coding genes, CNBG_4278 is the only one with a Kelch structural domain signature, as revealed by the superfamily database module of InterProScan. This domain is present in glyoxal and galactose oxidases, enzymes that interact with and modify carbohydrates (38). The CNBG_4278 ortholog in C. neoformans (CNAG_05595) was found in extracellular vesicles (EVs) known as “virulence bags” (39, 40), as well as in the cryptococcal secretome (41). Moreover, this gene was found to be differentially expressed in mutants for kinases that govern cell wall integrity (42). Hence, CNBG_4278 was characterized, hereafter described as Kelch repeat-containing protein 1 (Krp1). Despite the presence of a structural domain characteristic of Kelch domain-containing proteins, C. gattii Krp1 does not share similarity with these enzymes, not even with Saccharomyces cerevisiae and Candida albicans Kelch domain-containing protein (Kel1p) (Fig. S1).

10.1128/mSphere.00023-18.1 FIG S1  Phylogenetic tree for galactose oxidases, glyoxal oxidases, and Krp1 orthologs. Galactose oxidase sequences (blue), glyoxal oxidases (green), and Kel1p and Krp1 orthologs (pink) are shown. Kel1p from S. cerevisiae is identified by EIW10064.1, and Kel1p from C. albicans is identified by XP719052. Accession numbers in GenBank are listed at the beginning of each operational taxonomic unit (OTU). The full genus and species names of source organisms can be obtained from the respective GenBank entries. UniProt identifier Q01745 was used in the case of Fusarium graminarium GalOX. Download FIG S1, TIF file, 2.1 MB. Copyright © 2018 Reuwsaat et al. 2018 Reuwsaat et al. This content is distributed under the terms of the Creative Commons Attribution 4.0 International license.

10.1128/mSphere.00023-18.5 TABLE S1  Predicted C. gattii mannoproteins. Download TABLE S1, DOCX file, 0.1 MB. Copyright © 2018 Reuwsaat et al. 2018 Reuwsaat et al. This content is distributed under the terms of the Creative Commons Attribution 4.0 International license.

### Krp1 is antigenic but not essential for C. gattii virulence *in vivo.*

**FIG 1:**
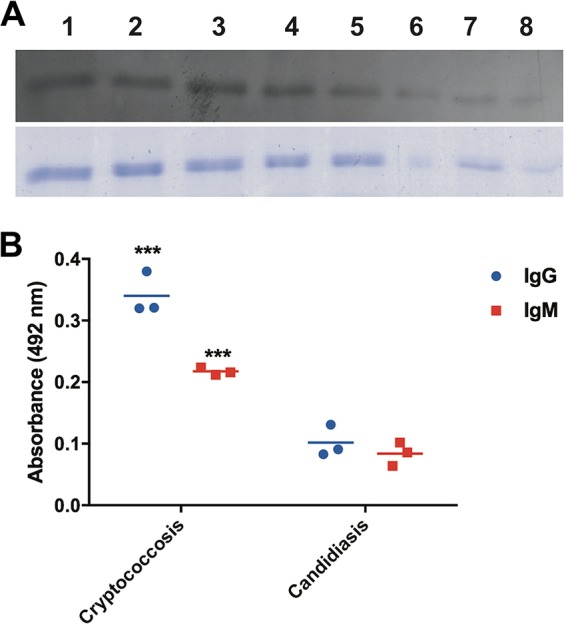
Recombinant Krp1 is antigenic. (A) Western blot. Purified recombinant truncated Krp1 (rKrp1t) expressed in E. coli was serially diluted (1:2) and probed with pooled sera from cryptococcal patients (top panel). SDS-PAGE mirror gel (bottom panel). (B) ELISA. One microgram of purified rKrp1t was probed with sera from patients with cryptococcosis and candidiasis. Results were analyzed by unpaired *t* tests. OD readings to determine IgG and IgM are shown. Values that are significantly different (*P* < 0.001) by *t* test are indicated by three asterisks.

Considering that the Krp1 ortholog from C. neoformans was found in the secretome, it was hypothesized that such a protein could elicit an immune response in the host. The recombinant Krp1 was produced in Escherichia coli to evaluate the antigenicity of the predicted mannoprotein Krp1. As the expression of recombinant full-length fungal mannoproteins generally displayed a low yield when expressed in prokaryotic systems (43), a truncated form (rKrp1t) was generated that lacked the signal peptide (first 20 amino acids) and the predicted GPI anchoring signal (last 30 amino acids). Due to the presence of a His_6_ tag in the carboxy terminus, the recombinant protein was purified by cobalt affinity chromatography. The purified rKrp1t was serially diluted and submitted to Western blotting using pooled sera from patients with cryptococcosis. A strong recognition signal could be detected with all dilutions of rKrp1t, confirming the Krp1 antigenicity (Fig. 1A). Also, an enzyme-linked immunosorbent assay (ELISA) was performed to confirm the recognition specificity of the recombinant Krp1 using sera from patients infected by other fungal pathogens. Optical density (OD) readings using sera from cryptococcosis patients were at least five times higher (IgG) or three times higher (IgM) than those obtained with sera from patients with candidiasis (Fig. 1B). These results suggest that the predicted mannoprotein is produced during infection and can be recognized by the host.

**FIG 2:**
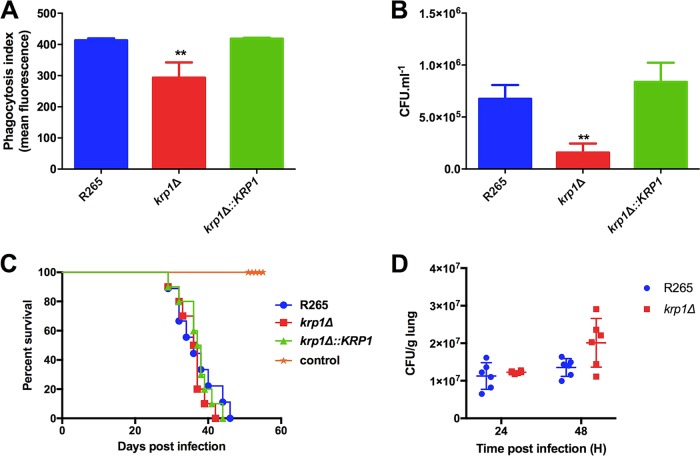
Deletion of the *KRP1* gene alters the yeast phagocytosis rate but is not necessary for the full virulence of C. gattii. (A) Phagocytosis index was assessed by flow cytometry after 2 h of interaction of FITC-labeled WT, *krp1*Δ, and *krp1*Δ::*KRP1* strains of cryptococcal cells with J774.A1 macrophages. (B) After 2 h of interaction of WT C. gattii, *krp1*Δ, and *krp1*Δ::*KRP1* cells with J774.A1 cells, murine cells were lysed, and the numbers of CFU per milliliter were determined. Data are shown as the means plus standard deviations (SD) for three biological replicates. One-way analysis of variance (ANOVA) followed by posthoc Dunnett test was performed. Values that are significantly different (*P* < 0.01) from the value for the wild-type strain (R265) are indicated by two asterisks. (C) Virulence assay in an intranasal inhalation infection model using BALB/c mice. (D) Fungal load in mouse lungs collected 24 or 48 h postinfection with C. gattii WT and *krp1*Δ cells.

To understand the role of Krp1 in C. gattii virulence, *krp1*Δ null mutant and *krp1*Δ::*KRP1* complemented mutant strains were constructed (Fig. S2). As macrophages are the host’s first line of defense against cryptococcal cells, the outcome of wild-type (WT), mutant, and complemented strains from *in vitro* interactions with phorbol myristate acetate (PMA)-activated J774.A1 macrophages were evaluated. The phagocytosis index of *krp1*Δ cells was lower than those of the WT and complemented strains as seen after 2 h of coincubation either by flow cytometry and by cryptococcal CFU determination analyses (Fig. 2A and B), suggesting that the absence of Krp1 altered the association of cryptococcal cells with macrophages, at least at the early stages of interaction. This was also confirmed by a Giemsa assay (Fig. S3). In order to evaluate whether the lower phagocytosis index of *krp1*Δ cells would reflect an altered virulence, BALB/c mice were intranasally infected with the WT, mutant, and complemented strains. No significant differences in the survival of the mice infected with the different C. gattii strains were observed (Fig. 2C), suggesting that Krp1 is not fundamental for cryptococcal virulence. In order to evaluate whether the reduced phagocytosis index determined *in vitro* would be observed *in vivo*, histopathological analysis was conducted using lungs of mice infected with WT and mutant strains. We did not detect a statistically significant difference in the lung fungal burdens from mice infected with the WT or *krp1Δ* strains, either at 24 or 48 h postinfection (Fig. 2D). In addition, histopathological analyses were conducted using lungs collected from mice infected with the WT or *krp1Δ* strain. After 24 h of infection, lungs presented mild neutrophilic and lymphohistiocytic inflammatory infiltrate, independent of the strain used to infect the mice (Fig. S4). These results confirm that the absence of Krp1 did not altered the pathological properties of cryptococcal cells.

10.1128/mSphere.00023-18.2 FIG S2  Deletion and complementation of *KRP1* gene for functional analysis. (A) Scheme for the construction of null mutant strain (Δ) is shown. All primers and the cleavage site of HindIII restriction enzyme are indicated. NAT, nourseothricin marker. (B) Southern blot analysis. Genomic DNA (10 µg) from WT (lane 1), *krp1*Δ (lane 2), and *krp1*Δ::*KRP1* (lane 3) strains were digested with HindIII restriction enzyme. The 3′ gene flank was used as the probe in Southern hybridization. Numbers on the left indicate the hybridization signal sizes based on the position of the molecular size marker (in base pairs [pb]). (C) Semiquantitative RT-PCR with cDNA from WT (lane 1), *krp1*Δ (lane 2), and *krp1*Δ::*KRP1* (lane 3) strains. Lane 4, negative control of the PCR; lane M, molecular size marker 1 kb plus DNA ladder, indicated in base pairs (pb) to the left of the gel. The top panel shows the amplification of *KRP1* transcripts, and the bottom panel shows the amplification of actin transcripts (*ACT1*). cDNAs were synthesized from RNA isolated from fungal cells grown in YPD for 24 h. Download FIG S2, JPG file, 2.8 MB. Copyright © 2018 Reuwsaat et al. 2018 Reuwsaat et al. This content is distributed under the terms of the Creative Commons Attribution 4.0 International license.

10.1128/mSphere.00023-18.3 FIG S3  Deletion of the *KRP1* gene alters the yeast phagocytosis rate but is not necessary for the full virulence of C. gattii. The phagocytosis index was assessed by Giemsa staining of PMA-activated J774.A1 macrophages after 2 h of interaction with WT, *krp1*Δ, and *krp1*Δ::*KRP1* strains of cryptococcal cells. One-way ANOVA followed by posthoc Dunnett test was performed. **, *P* < 0.01. Download FIG S3, TIF file, 0.1 MB. Copyright © 2018 Reuwsaat et al. 2018 Reuwsaat et al. This content is distributed under the terms of the Creative Commons Attribution 4.0 International license.

10.1128/mSphere.00023-18.4 FIG S4  Krp1 absence did not alter histopathology in mouse lung. Hematoxylin and eosin (HE) staining of lung sections collected after 24 h of infection with C. gattii R265 (top) and *krp1*Δ (bottom) strains. Magnification, ×400. Download FIG S4, JPG file, 1.4 MB. Copyright © 2018 Reuwsaat et al. 2018 Reuwsaat et al. This content is distributed under the terms of the Creative Commons Attribution 4.0 International license.

### Krp1 absence affects the C. gattii cell wall architecture.

**FIG 3:**
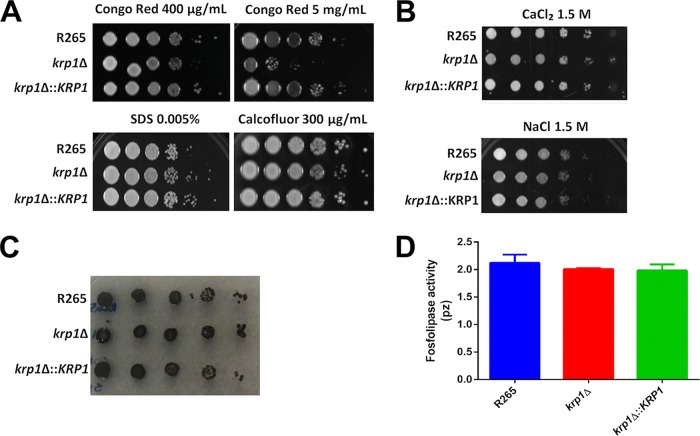
The absence of Krp1 led to defects in the cell wall but does not influence C. gattii cell wall-related virulence factors. (A and B) Growth test was performed by plating 3 µl of 10-fold serially diluted suspension of WT, *krp1*Δ, and *krp1*Δ::*KRP1* strains onto YPD agar supplemented with cell wall stressors (Congo red, SDS, and Calcofluor white) (A) or high-osmolarity stressors (CaCl_2_ and NaCl) (B) as indicated. (C and D) In addition, these dilutions were also spotted onto minimal medium agar supplemented with l-DOPA to evaluate melanization (C) and agar containing egg yolk emulsion to evaluate phospholipase activity (D).

To further investigate the lower phagocytosis index of *krp1*Δ mutant cells in the early stages of the interaction with macrophages, cell wall alterations that would influence cryptococcal recognition by the host were analyzed. *krp1*Δ cells displayed impaired growth in the presence of high doses of Congo red (Fig. 3A), a dye that may interact with nascent glucan chains and disrupts the balanced cell wall polymerization and crystallization (44). However, all strains grew similarly in the presence of the membrane stressor sodium dodecyl sulfate (SDS) and Calcofluor white (Fig. 3A), which interacts with nascent chitin chains (44, 45). Also, no growth differences were observed under high-osmolarity conditions (Fig. 3B). We also investigated whether the melanization process could be influenced by Krp1 knockout, as cell wall defects influence the deposition of melanin (32). No differences in melanin synthesis and/or deposition could be observed in WT, mutant, and complemented strains cultured in the presence of l-3,4-dihydroxyphenylalanine (l-DOPA) (Fig. 3C). Moreover, the levels of phospholipase B1 activity, an essential virulence factor from *Cryptococcus*, associated with the cell wall-promoting fungal membrane biogenesis and remodeling (46), were also similar in the strains tested (Fig. 3D).

**FIG 4:**
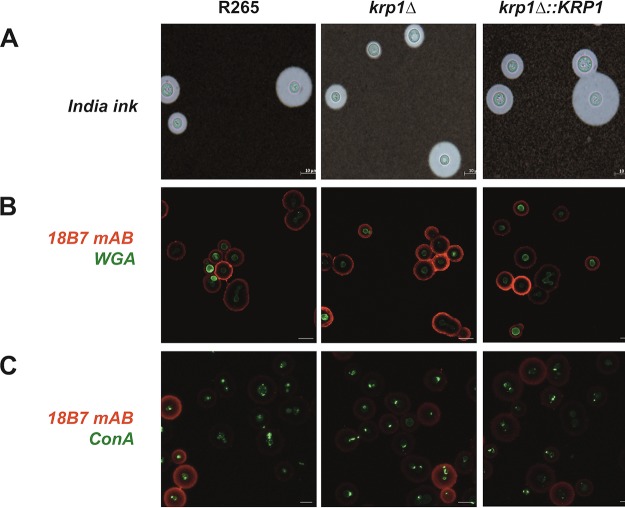
The absence of Krp1 does not alter capsule production nor chitooligomers and mannose distribution in cryptococcal cell surface. (A) India ink staining of C. gattii cells grown under capsule-induced conditions. Bars, 10 µm. (B and C) C. gattii cells were grown under capsule-induced conditions supplemented with Congo red (625 µg/ml) and labeled with the anti-GXM monoclonal antibody 18B7 to detect capsule (red) and with WGA (green) (B) to detect chitooligomeric structures. Alternatively, such cells were also labeled with the lectin ConA (green) (C) to detect mannosyl residues. Images were captured by a fluorescence confocal microscope. Micrographs were taken at a magnification of ×630.

The misdistribution of some cell wall components, including chitooligomers, was shown to be involved in the alteration of the phagocytosis index of C. neoformans cells (47). The distribution of some cell wall components was evaluated in the WT, *krp1*Δ, and *krp1*Δ::*KRP1* strains cultured under capsule-inducing conditions. As observed by India ink counterstaining, all strains produced similar capsules with regard to morphology and size (Fig. 4A). Using fluorescence-labeled versions of wheat germ agglutinin (WGA) and concanavalin A (ConA) lectins, the distribution of chitooligomers and α-d-mannosyl groups, respectively, was evaluated. There were no significant differences in the cell wall WGA or ConA staining pattern among the tested strains (Fig. 4B and C). In addition, probing such mutants with the anti-GXM monoclonal antibody (MAb) 18B7 also revealed a similar staining pattern (Fig. 4B and C). Together, these results indicate that Krp1 is important for glucan structuration in the C. gattii cell wall but does not influence the distribution of important cell wall components and capsule size.

### *KRP1* disruption influences interactions of capsular polysaccharides.

**FIG 5:**
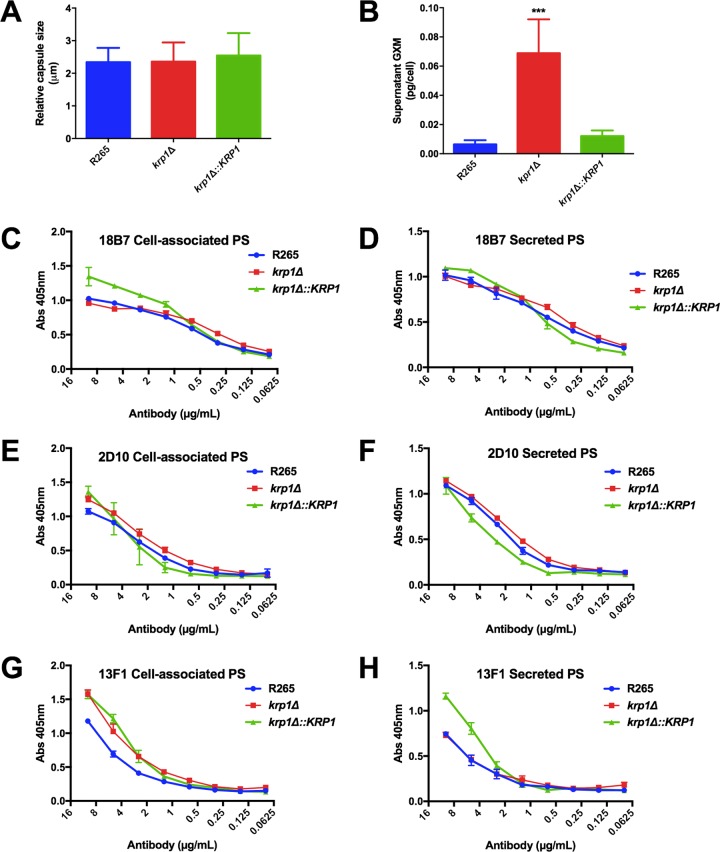
*KRP1* disruption does not affect capsule size or GXM serological proprieties but causes secretion into the extracellular space. (A) Capsule size was measured as the ratio of capsule size to cell diameter from at least 50 cells. (B) Secreted polysaccharides were quantified by ELISA with anti-GXM 18B7. Data are shown as the means plus standard deviations (SD) (error bars) for three biological replicates. One-way ANOVA followed by posthoc Dunnett test was performed. Values that are significantly different (*P* < 0.001) from the value for the wild-type strain (R265) are indicated by three asterisks. (C to H) Serological tests with MAbs 18B7, 2D10, and 13F1 of cell-associated and secreted polysaccharides (PS) from WT and *krp1* null or complemented mutants. Data are shown as the means ± SD for three biological replicates.

As no differences in the distribution of cell wall components were observed that could explain the low levels of *krp1*Δ phagocytosis by macrophages, the biophysical and serological proprieties of the antiphagocytic capsule components were determined. As previously demonstrated, capsule size did not differ among the strains analyzed (Fig. 5A). However, deletion of the *KRP1* gene altered the amount of extracellular GXM, as higher levels of the polysaccharide were detected in *krp1*Δ culture supernatants (Fig. 5B). To further investigate possible alterations in GXM recovered from the different strains that could explain the higher levels of GXM not attached to the cell wall, the serological reactivity of cell-associated and extracellular polysaccharide fractions was evaluated using MAbs that recognize distinct epitopes in the GXM molecule. No significant differences in immunoreactivity to distinct antibodies (MAbs 18B7, 2D10, and 13F1) were found between cell-associated and released GXM recovered from the strains analyzed (Fig. 5C to H).

**FIG 6:**
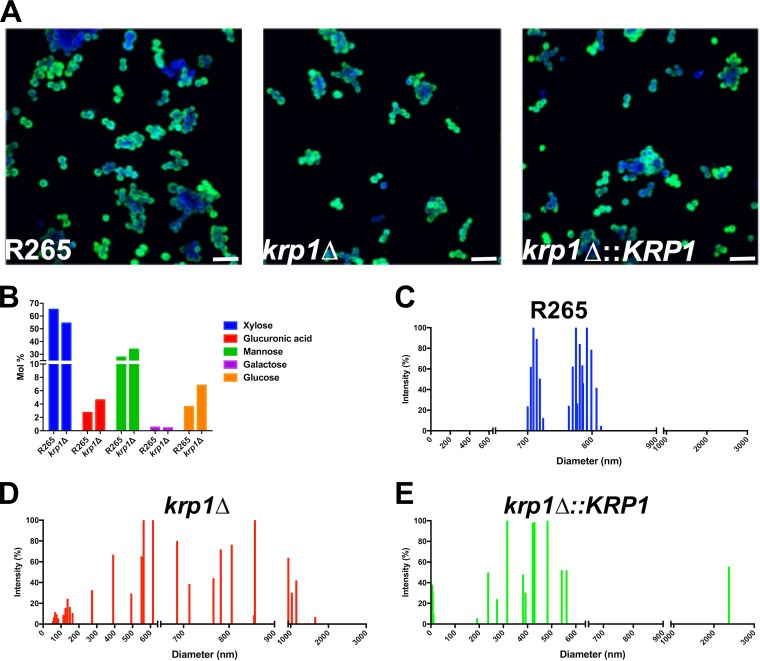
Morphological and structural proprieties of cell-associated and secreted polysaccharides from C. gattii strains. (A) Capsule transfer assay. C. neoformans *cap67* cells were grown in YPD medium and incubated with secreted polysaccharides from WT and mutant C. gattii strains. For confocal microscopy, C. neoformans cells were labeled with Calcofluor white (blue) and MAb 18B7 (green). Bars, 20 µm. (B) Cell-associated polysaccharides were isolated from WT and *krp1*Δ cells, and their elemental composition was determined by GC-MS. (C to E) Cell-associated polysaccharide molecular dimension determination of WT, *krp1*Δ, and *krp1*Δ::*KRP1* strains using DLS analysis. The cells were cultured in minimal medium for 72 h, and the cell-associated polysaccharides were extracted using DMSO. Each graph displays the range of polysaccharide fiber sizes representative of three independent analyses.

In order to evaluate possible defects of *krp1*Δ-produced polysaccharides in cell wall attachment, the morphological aspects of GXM incorporation by acapsular C. neoformans *cap67* cells were evaluated by fluorescence microscopy (Fig. 6A). Extracellular polysaccharides were purified from WT, *krp1*Δ, and *krp1*Δ::*KRP1* strains cultured under capsule-inducing conditions and fed to C. neoformans *cap67* cells. Fluorescence microscopy employing the anti-GXM MAb 18B7 did not reveal significant differences in the incorporation of GXM produced by the mutant strain and the WT (Fig. 6A). These results are in agreement with no significant differences among the elemental composition of capsular GXM produced by *krp1*Δ and WT cells (Fig. 6B). As cells lacking Krp1 displayed altered GXM concentration in secreted polysaccharides, it was hypothesized that Krp1 could also influence the physical characteristics of polysaccharide fibers. Dynamic light scattering (DLS) measurements were used to determine the size distribution of polysaccharide fibers recovered from WT, *krp1*Δ, and *krp1*Δ::*KRP1* strains. While WT cell-associated polysaccharides present a clear bimodal distribution size, ranging from 700 to 820 nm (Fig. 6C), the absence of *KRP1* disorganized the capsule polysaccharide size distribution, leading to a scattered pattern (Fig. 6D). It is noteworthy that such a phenotype was not completely restored by complementation of the WT gene (Fig. 6E), possibly due to defects in the proper expression of the complementation *KRP1* cassette, which is integrated in an ectopic locus.

**FIG 7:**
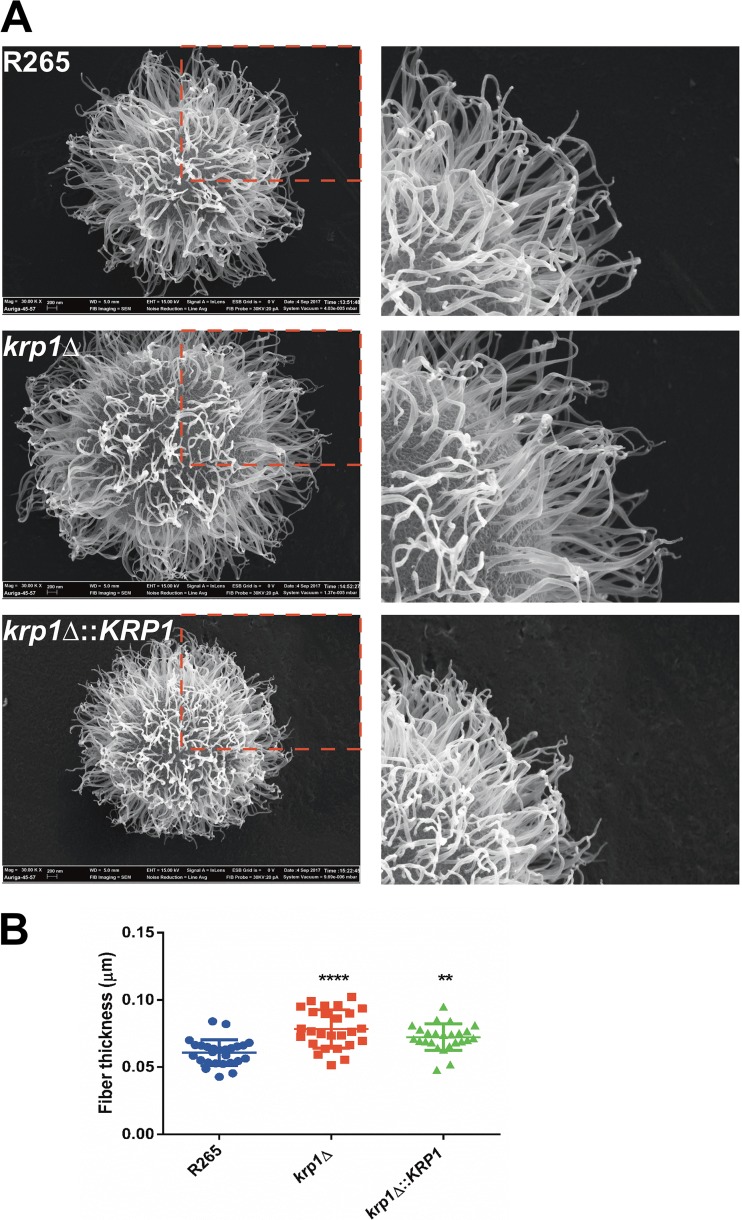
Morphological analysis of WT and Krp1 mutants. (A) FEG-SEM analysis of representative cells belonging to the different genotypes. The right panels represent magnified views of the capsular structures outlined by red broken lines in the left panels. (B) Fiber thickness measured from 20 to 30 cells of WT, *krp1*Δ, and *krp1*Δ::*KRP1* strains. One-way ANOVA followed by posthoc Dunnett test was performed. Values that are significantly different from the value for the wild type (R265) are indicated by asterisks as follows: ****, *P* < 0.0001; **, *P* < 0.01.

To further explore such differences, field emission gun scanning electron microscopy (FEG-SEM) analysis of WT, *krp1*Δ, and *krp1*Δ::*KRP1* strains was performed. A dense array of capsular fibers could be observed in all strains analyzed (Fig. 7A). However, the fibers in the *krp1*Δ cell surface tended to be thicker than those produced by the WT strain (*P* < 0.0001; Fig. 7B). This phenotype was partially reconstituted in the complemented strain (*P* < 0.01; Fig. 7B).

**FIG 8:**
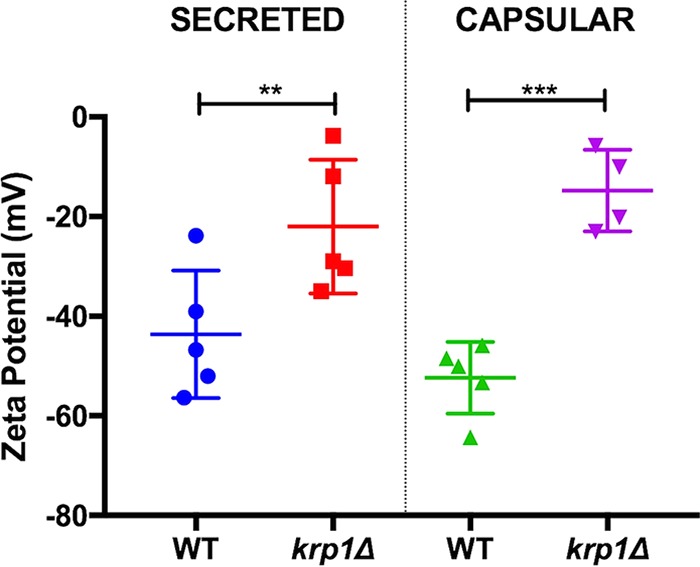
The absence of Krp1 led to altered net charge in capsular polysaccharides. Cells were cultured in minimal medium for 72 h. The cell-associated and secreted polysaccharides were isolated, and their net charge was determined using a zeta potential analyzer. Student’s *t* test was performed to evaluate statistical differences (**, *P* < 0.01; ***, *P* < 0.001).

In order to gain insights from the physical-chemical interactions that would be altered in the cells lacking *KRP1*, we performed zeta potential determinations using polysaccharide (PS) isolated from cells, as well from the supernatant. We observed an increased zeta potential in both PS fractions recovered from cells lacking Krp1, suggesting that this mannoprotein is involved in the proper maintenance of capsular charge (Fig. 8). Altogether, these results suggest that Krp1 may prompt interaction of polysaccharides of the C. gattii capsule.

## DISCUSSION

Mannoproteins are important structural constituents of fungal cell walls (48) and components of the cryptococcal polysaccharide capsule (14). Despite some evidence of the immunogenicity of these proteins (33, 49), little is known about their role in capsule structuring. Bioinformatic analysis of the predicted proteomes of two clinically important *Cryptococcus* species identified 43 predicted mannoproteins encoded by C. neoformans H99 genes. This is in accordance with a previous report that found 53 serine-threonine-rich proteins containing a GPI anchor and signal peptide in the C. neoformans var. neoformans JEC21 predicted proteome (33). For C. gattii, a total of 36 mannoproteins were predicted. Most of the predicted mannoproteins in both species are annotated as hypothetical proteins, with a small proportion of them spanning conserved domains in their sequences. Only two predicted C. gattii mannoproteins did not display the C. neoformans ortholog, because of the loss of the GPI anchor (see Table S1 in the supplemental material). Similarly, four predicted C. neoformans mannoproteins did not have the C. gattii ortholog identified as a mannoprotein due to the loss of the signal peptide or GPI anchor (Table S2), including MP88 and *CDA1*. Also, C. neoformans genes encode five mannoproteins that do not have an ortholog in C. gattii (Table S2). As expected, more than 40% of the predicted mannoproteins do not possess conserved domains, and the majority of the mannoproteins with conserved domains have evidence of carbohydrate modifications (Tables S1 and S2).

10.1128/mSphere.00023-18.6 TABLE S2  Predicted C. neoformans mannoproteins. Download TABLE S2, DOCX file, 0.1 MB. Copyright © 2018 Reuwsaat et al. 2018 Reuwsaat et al. This content is distributed under the terms of the Creative Commons Attribution 4.0 International license.

The predicted mannoprotein characterized in this study (Krp1) presents a predicted folding found in Kelch repeats, a domain usually associated with galactose or glyoxal oxidases (50, 51) and related to altered cell fusion and morphology in S. cerevisiae and C. albicans (52). However, our analysis did not reveal conservation among cryptococcal Krp1 and other Kelch repeat-containing proteins from diverse fungal species (Fig. S1). It is noteworthy that the Krp1 ortholog in C. neoformans (CNAG_05595) was detected in the secretome and extracellular vesicles (40, 41, 53). As proteins associated with capsule structure were detected in the extracellular space (54), it is possible that Krp1 could also be involved in this process.

Mannoproteins have a potential importance in immunity-based strategies to control cryptococcosis due to their role in T cells and the humoral induction response in the host (55). This study showed that the recombinant Krp1 expressed in E. coli, even without glycosylation, was recognized by sera from cryptococcosis patients. The presence of epitopes in the protein sequence irrespective of posttranslational modifications (PTMs) revealed by Western blot analysis is evidence that Krp1 could elicit an immune response. However, at this moment, it is not possible to evaluate whether the native Krp1 would elicit a more intense immune response. It is important to note that such characteristics could also be observed for other C. neoformans mannoproteins (56), suggesting the existence of an epitope set that would work without any PTMs. The recombinant Krp1 (rKrp1) was not fully recognized by sera from patients with candidiasis, an important feature for specific cryptococcal antigens that could serve for diagnosis. However, a more in-depth analysis must be performed with sera from patients infected with different pathogens to confirm the specificity of rKrp1 recognition as a cryptococcal antigen. Importantly, the serological reactivity of rKrp1 with patient sera suggests its production during infection.

Cells lacking the *KRP1* gene displayed impaired phagocytosis by macrophages, at least in the initial stages of coincubation. Cryptococcal uptake by phagocytes is dependent on the recognition of pathogen-associated molecular patterns (PAMPs) present in the yeast cell surface by pattern recognition receptors (PRRs) on host cells (57). Cryptococcal PAMPs include capsule- and cell wall-derived constituents as well as melanin (57). However, one of the several capsule functions is the capability to mask the PAMPs, reducing the recognition of fungal cells by macrophages (58). The first hypothesis for impaired phagocytosis of *krp1*Δ cells was a possible cell wall defect that could have modified the structuration of PAMPs. In line with these assumptions, null mutant cells were found to display higher sensitivity to the cell wall stressor Congo red, a dye that interacts with beta-glucan nascent chains and impairs the activity of assembly enzymes that connect chitin to it (45). As *krp1*Δ cells do not present variations in the distribution of surface components directly associated with host defense, the second hypothesis for its decreased rate of phagocytosis by macrophages was related to its capsule structure and assembly. Even without capsule length differences as measured by the penetration of India ink molecules, *krp1*Δ cells present much more GXM secreted in the culture supernatant. GXM is a known immunoregulatory molecule (58), and the reduced phagocytosis rate of *krp1*Δ cells could be associated with the higher GXM present in the supernatant, which in turn would modulate the activity of macrophages. We do not associate the lower phagocytosis of *krp1*Δ cells to PAMPs as mannose or chitooligomeric structures, as no differences could be observed in cells lacking the *KRP1* gene compared to WT cells. Taken together, these results suggest that higher GXM release modulates macrophage activity, at least during the initial interaction of cryptococci with host cells. Longer incubation periods would buffer this difference by the possible compensatory activity of other proteins. However, this hypothesis needs to be experimentally validated. It is important to note that Krp1 is possibly associated with the onset of infection, demonstrating that alterations in the surfaces of *krp1*Δ cells interfere with the steady activation of macrophages. Nonetheless, Krp1 is not important for C. gattii virulence in a murine model of cryptococcosis, which adds a layer of complexity to the function of mannoproteins.

The mechanisms by which capsular components associate with the cryptococcal cell wall in order to build the capsule are still under investigation, but the clear participation of glucans and chitosan has been described (59–62). The *Cryptococcus* cell wall is composed of β-1-3- and β-1-6-glucans, α-1,3-glucans, chitin, chitosan, melanin, glycoproteins and plasma membrane-derived glucosylceramides (63, 64). The innermost part is formed by parallel fibers composed of β-glucans, chitin, and when present, melanin. The outermost layer of the wall is mainly composed of α- and β-glucans, forming a more particulate network (65). Unlike other fungi, C. neoformans is composed of more molecules of β-1-6-glucans than β-1-3-glucans (18). Even without proving the direct contact of Krp1 with the cryptococcal capsule, there is evidence to suggest that Krp1 is involved in GXM fiber structuration to the cell wall. (i) Cells lacking Krp1 were hypersensitive to the β-glucan stressor Congo red. (ii) The GXM content of culture supernatants was altered due to the absence of Krp1. (iii) C. neoformans *cap67* cells, which have a Krp1 ortholog, were capable of binding extracellular polysaccharides released by C. gattii *krp1*Δ cells. (iv) Cell-associated cryptococcal polysaccharide diameter and thickness are influenced by the presence of Krp1. Polysaccharide thickness has recently been linked with capsular architecture and pathogenesis (66). Increased fiber thickness is indicative of higher levels of GXM self-aggregation, but other possibilities cannot be ruled out. For instance, altered carbohydrate composition (67) and or branching (68) can affect the thickness of cryptococcal polysaccharide fibers. On the basis of the relatively subtle differences between WT and mutant fibers, as well as of the partial reconstitution of the phenotype in complemented cells, it is still impossible to establish the reasons behind the altered fiber thickness. However, we believe that it is appropriate to propose that this parameter be included in the analysis of the multiple factors connecting capsular structure with pathogenesis in *Cryptococcus*.

The *Cryptococcus* cell wall matrix is structured by the activity of extracellular cross-linking enzymes that covalently bind carbohydrate polymers and glycoproteins (48) that prepare the structure for capsule attachment. The *Cryptococcus* capsule grows by enlargement of secreted polysaccharide molecules (67), usually secreted by extracellular vesicles, and aggregate in the presence of divalent cations (69). Also, the C. neoformans Krp1 ortholog was previously detected in the secretome and extracellular vesicles (39–41). Here, we report the role of Kpr1 in C. gattii capsule structuring, mainly by modulating the distribution of glucans in the yeast cell wall.

## MATERIALS AND METHODS

### Fungal strains, plasmids, and media.

The Cryptococcus gattii R265 strain used in this study was kindly provided by Wieland Meyer (The University of Sydney, Australia). Plasmid pDNORNAT, which contains the nourseothricin marker cassette, was previously constructed by our group (70). Plasmid pJAF15, which contains the hygromycin resistance marker cassette was a generous gift of Joseph Heitman (Duke University, Durham, NC, USA). The strains were maintained on YPD agar (2% dextrose, 2% peptone, 1% yeast extract, and 1.5% agar). YPD plates containing nourseothricin (100 µg/ml) were used to select C. gattii mannoprotein deletion transformants (*krp1*Δ), and YPD plates containing hygromycin (200 µg/ml) were used to select C. gattii mannoprotein reconstituted transformants (*krp1*Δ::*KRP1*).

### Bioinformatic analysis.

The predicted proteomes of C. gattii R265 and C. neoformans H99 were retrieved from the Broad Institute (71–73) and now available at the FungiDB database (http://fungidb.org/fungidb/). SignalP was used for signal peptide prediction (34), PredGPI was used for glycosylphosphatidylinositol (GPI) anchor prediction (35), and GlycoEP standard was used for O-glycosylation analysis (36). The search for conserved domains was performed using InterProScan (https://www.ebi.ac.uk/interpro/search/sequence-search). For phylogenetic inference, *KRP1* sequence (CNBG_4278) and its orthologs (CGBL_3040C, CNAG_05595, CNH_02380, and CNBL_2400) obtained from FungiDB (http://fungidb.org/fungidb/), were added to sequences of galactose oxidases and glyoxal oxidases described by Yin and colleagues (38). The sequence alignment was performed in MAFFT v7 server with default parameters. The evolutionary model for amino acid substitutions was determined by ProtTest v3.4.2. The tree was constructed with MEGA 6.0 and maximum likelihood method with a bootstrap of 1,000 replicates.

### Recombinant expression of Krp1.

For expression in E. coli, the modified coding sequence of the *KRP1* gene (without signal peptide and GPI anchor site, which refers to amino acids 21 to 381 of the primary sequence) was cloned into pET-23D(+) between the sites of BamHI and HindIII (Invitrogen Corp., Carlsbad, CA, USA). Cloning was confirmed by cleavage and DNA sequencing. For the expression of the recombinant mannoprotein, the E. coli BL21(DE3) strain was transformed with the p*LYS*s plasmid, and protein expression was induced with lactose (20 g/liter) for 3 h. Purification was conducted under denaturing conditions with HiTrap immobilized metal affinity chromatography (IMAC) (GE Healthcare Life Sciences) charged with 100 mM CoCl_2_. Buffers with increasing concentrations of imidazole (50 mM Na_2_HPO_4_, 300 mM NaCl, 8 M urea, and 10 to 500 mM imidazole) were used for elution of the recombinant protein from the IMAC column.

### Western blotting and ELISA with patient sera.

The serological properties of recombinant Krp1 were evaluated by Western blotting and ELISA using sera from individuals diagnosed with cryptococcosis. For Western blotting, the purified truncated recombinant Krp1 (rKrp1t) fraction was separated by SDS-PAGE, transferred to a polyvinylidene fluoride membrane, and probed with pooled sera from patients with cryptococcosis at a dilution of 1:10. Detection was performed using a Pierce ECL Western blotting substrate (Thermo Fisher Scientific) according to the manufacturer’s instructions using anti-human IgG conjugated to peroxidase. For ELISA, a total of 1 µg of purified rKrp1t was used to sensitize ELISA microplates (BD Falcon 3912) and probed with sera from patients with cryptococcosis and candidiasis for cross-reactivity evaluation at a dilution of 1:200. The IgG and IgM conjugates were quantified using ZyMax goat anti-human IgG H+L and horseradish peroxidase (HRP)-conjugated goat anti-human IgM secondary antibodies according to the manufacturer’s instructions.

### Disruption and complementation of *KRP1.*

Disruption of the *KRP1* gene was achieved by the Delsgate methodology (74). The 5′ and 3′ *KRP1* flanks (781 bp and 771 bp, respectively) were PCR amplified and purified from agarose gels (PureLink quick gel extraction kit; Invitrogen, Germany). *Double-joint* PCR with 1 ng of each fragment was carried out, resulting in a fragment of 1,552 bp. Approximately 200 ng of pDONRNAT and 100 ng of each PCR product were submitted to BP clonase reaction, according to the manufacturer’s instructions (Invitrogen, Carlsbad, CA). The product of this reaction was transformed into E. coli TG-2. After confirmation of the correct deletion construct, the plasmid was linearized with I-SceI prior to C. gattii biolistic transformation (75). The transformants were screened by colony PCR, and the deletion was confirmed by Southern blot and semiquantitative reverse transcription-PCR (RT-PCR) analysis. For complementation, a nearly 3.4-kb genomic PCR fragment containing the wild-type *KRP1* gene was cloned into the EcoRV site of pJAF15. The resulting plasmid was used for *krp1*Δ strain transformation. Genomic insertion of the complemented gene was confirmed by Southern blotting and semiquantitative RT-PCR analysis. The primers used in constructing these plasmids are listed in Table S3 in the supplemental material.

10.1128/mSphere.00023-18.7 TABLE S3  Primers used in the present work. Download TABLE S3, DOCX file, 0.1 MB. Copyright © 2018 Reuwsaat et al. 2018 Reuwsaat et al. This content is distributed under the terms of the Creative Commons Attribution 4.0 International license.

### Macrophage assays.

Phagocytosis assays were conducted to evaluate the susceptibility of the mutant strains to macrophage antifungal activity. One day before the phagocytosis test, an aliquot of 100,000 J774.A1 cells in DMEM (Dulbecco’s modified Eagle medium) supplemented with 10% fetal bovine serum (FBS) was seeded into 96-well culture plates and cultivated for 24 h at 37°C and 5% CO_2_. The C. gattii strains were inoculated into YPD and allowed to grow at 18 h at 30°C. Then, C. gattii cells were washed three times with phosphate-buffered saline (PBS) and counted. A total of 10^7^ cells of each strain were opsonized with antiglucuronoxylomannan (anti-GXM) monoclonal antibody (MAb) 18B7 (final concentration of 1 µg/ml) and incubated for 1 h at 37°C. At the same time, macrophage cells were washed once with warm PBS and incubated in FBS-free DMEM with 5 nM phorbol myristate acetate (PMA) for activation for 2 h. Then, macrophages were exposed to yeast cells at a ratio of 1:10 and incubated for 2 h at 37°C and 5% CO_2_. At the end of incubation, the wells were washed three times with warm PBS, the macrophage cells were lysed with sterile ice-cold water, and subsequently plated on YPD plates for CFU determination. For the Giemsa assay, at the end of interaction, macrophage cells were fixed with methanol and stained with Griess for 15 min at room temperature. Cells were visualized using a Zeiss Axiovert 200 inverted fluorescence microscope equipped with an AxioCam MRc camera (Carl Zeiss, Jena, Germany). The images were acquired using AxioVision Rel 4.8 software. For flow cytometry analysis, after opsonization, yeast cells were labeled with fluorescein isothiocyanate (FITC) (Sigma). Murine macrophage cells were cultured in a 12-well culture plate. After 2-h incubation, the wells were washed with warm PBS, and trypan blue was added to each well to quench the fluorescence of labeled yeast attached to the outer membranes of the macrophages. Macrophages were detached from the plate using a cell scraper and analyzed by flow cytometry (Millipore Guava software). The phagocytosis index was determined as the ratio of internalized cryptococcal cells to the number of macrophage cells.

### Virulence assays.

Virulence studies were conducted according to a previously described intranasal inhalation infection model (76, 77) using 10 female BALB/c mice (approximately 4 weeks old) for each strain. Mice were infected with 10^5^ yeast cells suspended in 50 µl of PBS and monitored daily. Kaplan-Meier analysis of survival was performed to evaluate survival differences. For determination of the lung fungal burden, mice (*n* = 6) were infected with 10^7^ yeast cells. After 24 and 48 postinfection, the animals were euthanized, and the lungs were aseptically excised. The tissues were homogenized in PBS. After removal of host cell debris, the resulting suspensions were plated on YPD for CFU determination.

### Histopathology.

The lungs of mice infected with wild-type (WT) and *krp1*Δ cells were aseptically collected 24 h postinfection and fixed in 10% neutral buffered formalin. All lungs were then embedded in paraffin, cut into 5-µm-thick slices, and stained with hematoxylin and eosin. All analyses were by Axys Análises Diagnósticos Veterinário (Porto Alegre, Brazil). All slides were examined by light microscopy.

### Phenotypic characterization assays.

For phenotypic characterization, WT, null, and complemented strains were grown on YPD medium for 16 h, washed with PBS, and adjusted to a cell density of 10^7^ cells/ml. The cell suspensions were serially diluted 10-fold, and 3 µl of each dilution was spotted onto YPD agar supplemented with the cell wall stressor Congo red (400 µg/ml and 5 mg/ml), Calcofluor white (300 µg/ml), or SDS (0.005%) (45) and with the salts NaCl (1.5 M) and CaCl_2_ (1.5 M) (78). The plates were incubated for 2 days at 30°C and photographed. The solid melanization test was performed as described above, mixing 10 ml of 2× minimal medium (2 g/liter l-asparagine, 1 g/liter MgSO_4 _⋅ 7H_2_O, 6 g/liter KH_2_PO_4_, 2 g/liter thiamine, and 2 mM l-3,4-dihydroxyphenylalanine [l-DOPA]) with 10 ml of 2% agar-water per plate. For the phospholipase test, cells of all strains were spotted in agar containing egg yolk emulsion at a concentration of 8%. After 96 h of incubation at 30°C, the phospholipase activity (pz) was measured as the ratio of the colony diameter by the precipitation zone generated.

### Microscopy.

Cell surface morphology was analyzed after incubation of yeast cells with Calcofluor white, the monoclonal antibody 18B7 (79), wheat germ agglutinin (WGA), and concanavalin A (ConA). These probes were used to visualize cell wall chitin (Calcofluor white), GXM (MAb 18B7), chitooligomers (WGA), and α-d-mannosyl groups (ConA) by confocal microscopy following a previously described protocol (62). Briefly, 10^6^ cells were grown in DMEM for 72 h at 37°C and 5% CO_2_. After incubation, cells were fixed in 4% paraformaldehyde and washed three times with PBS. The concentrations of the probes used in this study were 5 µg/ml WGA, 5 µg/ml Calcofluor white, 10 µg/ml MAb 18B7, and 10 µg/ml ConA. The incubations were performed individually for 30 min at 37°C. After each incubation, cells were washed three times with PBS and analyzed with a confocal microscope FV1000, in the Electron Microscopy Center (CME) of the Universidade Federal do Rio Grande do Sul (UFRGS). For scanning electron microscopy, 10^6^ cells were grown in minimal medium for 72 h at 30°C and 200 rpm. Sample preparation was conducted as described previously (80), and the capsular structures were visualized with an Auriga field emission gun scanning electron microscopy (FEG-SEM) microscope (Zeiss, Germany). The thickness of WT and *KRP1* mutant fibers was measured in 20 to 30 cells with the ImageJ software as previously described (66).

### GXM purification and capsular transfer assay.

GXM was isolated from culture supernatant by ultrafiltration as previously described (69). Cellular polysaccharides were extracted with dimethyl sulfoxide (DMSO), following protocols that were established for efficient removal of GXM from C. neoformans cells (81). For capsular transfer assay, the C. neoformans acapsular *cap67* strain was used as the capsule acceptor. Briefly, 5 × 10^6^ cells/ml were incubated with purified GXM (10 µg/ml in PBS) for 1 h at room temperature followed by extensive washing (59). Cells were stained with MAb 18B7 and Calcofluor white as described above.

### Capsule size, GXM quantification, and serological analysis of polysaccharide fractions.

For capsule size measurement, WT, null, and complemented strains were grown on YPD medium for 16 h and washed with PBS, and 10^6^ cells were incubated in DMEM for 72 h at 37°C and 5% CO_2_. After incubation, cells were fixed in 4% paraformaldehyde and washed three times with PBS. C. gattii cells were placed on glass slides and mixed with similar volumes of India ink. Capsule sizes, defined as the distances between the cell wall and the outer border of the capsule in India ink-stained yeast cells, were determined using ImageJ software (version 1.33), elaborated and provided by National Institutes of Health (NIH) (http://rsb.info.nih.gov/ij/). Cell diameters were determined using the same software. The final measurements were presented as ratios of capsule size to cell diameter. Secreted polysaccharides were quantified by ELISA for specific GXM detection (82), and cellular polysaccharides were quantified by the phenol-sulfuric acid method for total carbohydrate determination (83). The serological analysis of polysaccharide fractions from WT and mutant cells was determined by ELISA with different mouse monoclonal antibodies to GXM (MAbs 2D10 and 13F1 [IgM] and 18B7 [IgG]) as previously described (69, 82).

### Capsule composition and dynamic light scattering analysis.

Glycosyl composition analysis was performed by combined gas chromatography-mass spectrometry (GC-MS) of the per-*O*-trimethylsilyl (TMS) derivatives of the monosaccharide methyl glycosides produced from the sample by acidic methanolysis as described previously (84). The dimensions of polysaccharides were determined by dynamic light scattering (DLS) as described by Frases and colleagues (67).

### Zeta potential determination.

The zeta potential, particle mobility, and shift frequency of cell-associated and secreted PS samples were calculated in a zeta potential analyzer (ZetaPlus; Brookhaven Instruments Corp., Holtsville, NY), as previously described (9).

### Ethics statement.

The use of animals in this work was performed with approval of the Universidade Federal do Rio Grande do Sul Ethics Committee for Use of Animals (CEUA 30936). Mice were housed in groups of four and kept in filtered top ventilated cages with food and water *ad libitum*. The animals were cared for according to the Brazilian National Council for Animal Experimentation Control (CONCEA) guidelines. All efforts to minimize animal suffering were made. Before infection assays, mice were intraperitoneally anesthetized with ketamine (100 mg/kg of body weight) and xylazine (16 mg/kg). Mice were monitored twice daily for any signs of suffering, defined by weight loss, weakness, or inability to obtain food or water. At the first signs of suffering, mice were euthanized with an overdose of thiopental (140 mg/kg) and lidocaine (10 mg/kg). The utilization of patients’ sera was approved by UFRGS Ethics Committee (CEP 19812). Informed consent was obtained from all participants.
