# Supplementary figures and images for: A Predicted Mannoprotein Participates in Cryptococcus gattii Capsular Structure
Source: mSphere. 2018 Apr 25;3(2):e00023-18. doi: 10.1128/mSphere.00023-18 (PMC5917426; doi:10.1128/mSphere.00023-18)

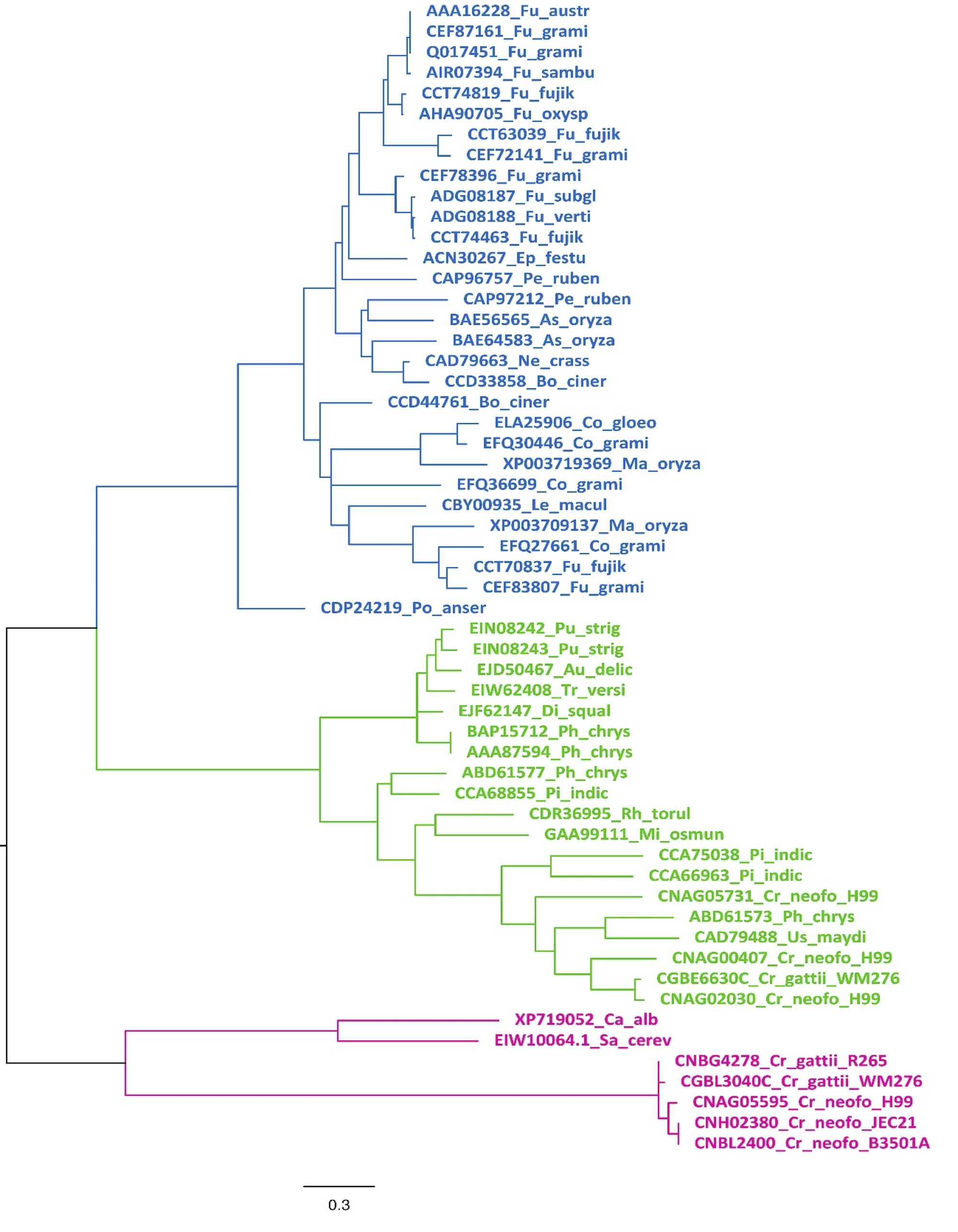

Supplement: FIG S1 [file sph002182524sf1.tif]

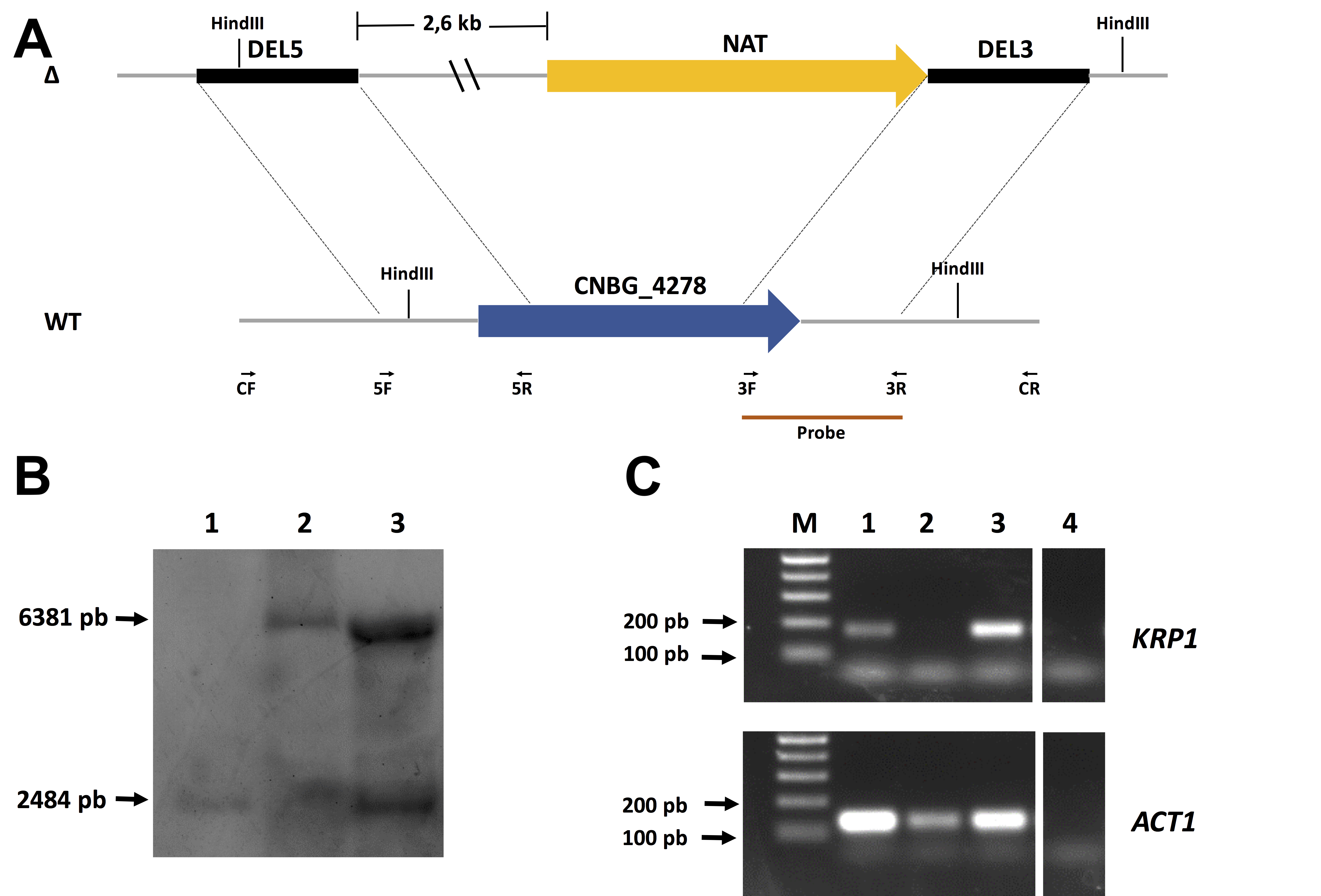

Supplement: FIG S2 [file sph002182524sf2.jpg]

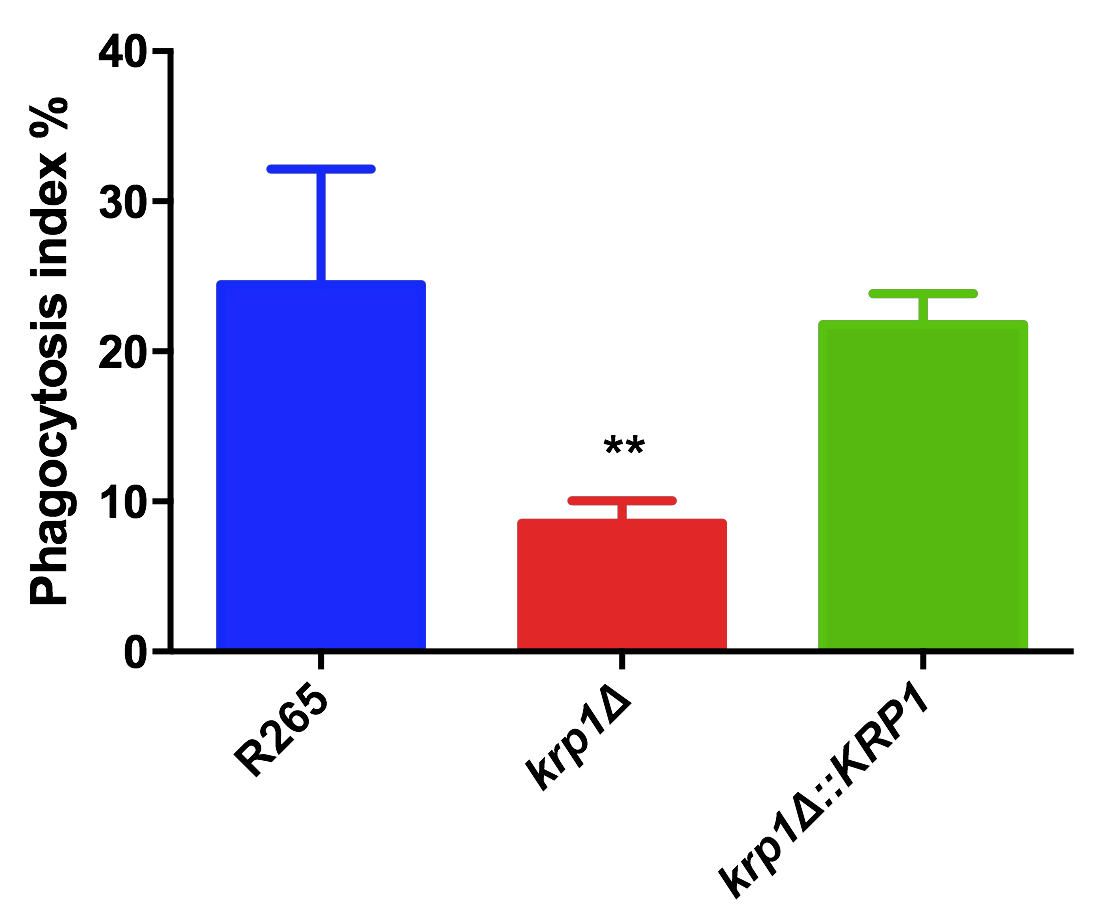

Supplement: FIG S3 [file sph002182524sf3.tif]

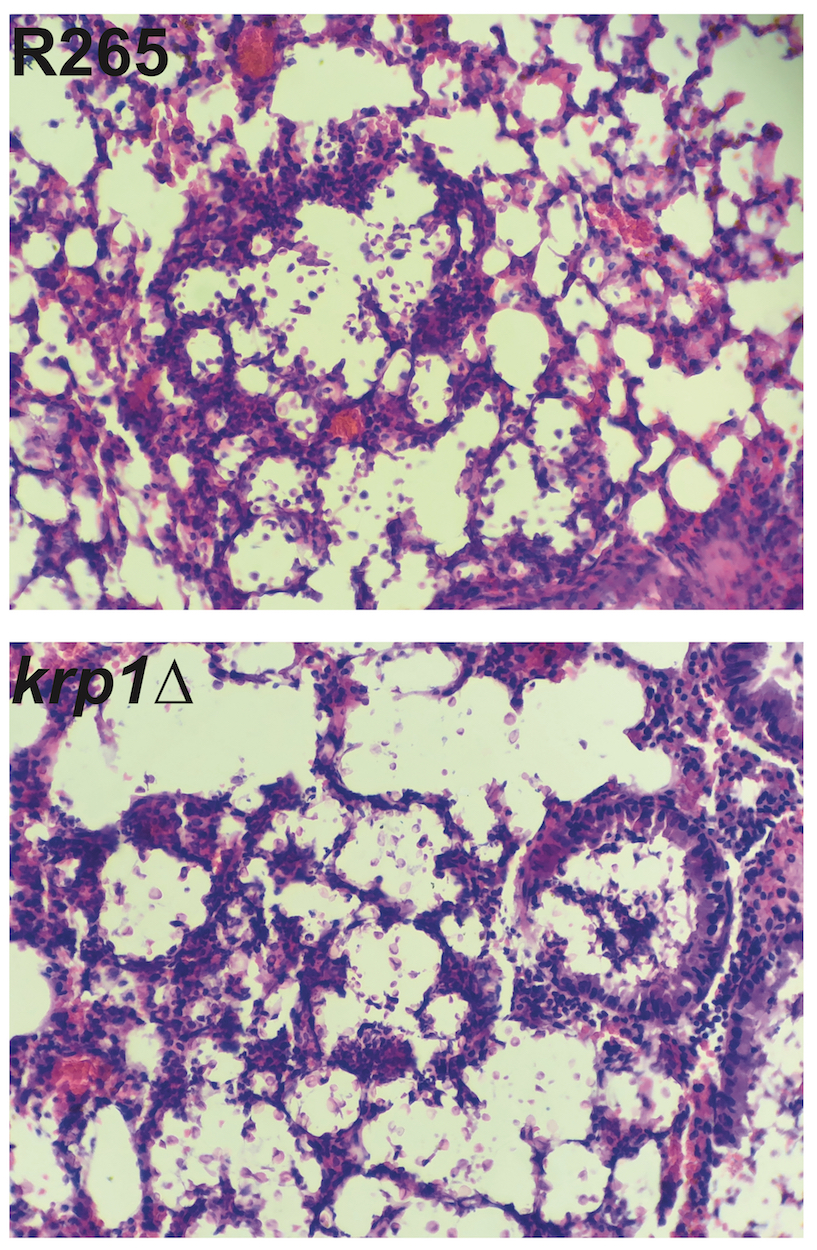

Supplement: FIG S4 [file sph002182524sf4.jpg]
