# Supplementary material for: A Predicted Mannoprotein Participates in Cryptococcus gattii Capsular Structure
Source: mSphere. 2018 Apr 25;3(2):e00023-18. doi: 10.1128/mSphere.00023-18 (PMC5917426; doi:10.1128/mSphere.00023-18)
Supplement: TABLE S1 [file sph002182524st1.docx]

**Table S1. *C. gattii* predicted mannoproteins**

| *C. gattii* predicted mannoproteins **^A^** | Annotation | Domain prediction **^B^** | Conservation (Coverage/Identity) **^C^** |
| --- | --- | --- | --- |
| **CNBG_4278** | Hypothetical | Kelch | - |
| **CNBG_0310** | Hypothetical | None | NSSF**^D^** |
| **CNBG_0972** | Hypothetical | None | 67/27 |
| **CNBG_1017** | Endopeptidase | Aspartic peptidase A1 | 25/35 |
| **CNBG_1499** | Glyoxal oxidase | Galactose/glyoxal oxidase | 47/25 |
| **CNBG_1575** | Hypothetical | None | 7/24 |
| **CNBG_2381** | Hypothetical | None | 7/36 |
| **CNBG_2705** | Hypothetical | Kre9/Knh1 family | 10/42 |
| **CNBG_3416** | Hypothetical | None | 8/35 |
| **CNBG_4403** | Endo-1,3(4)-β-glucanase | Glucanase | NSSF |
| **CNBG_4430** | Hypothetical | None | 2/50 |
| **CNBG_4538** | Hypothetical | CFEM domain | 3/42 |
| **CNBG_4742**^I^ | Glycoprotein | None | 3/53 |
| **CNBG_4814** | Hypothetical | Glycoside hydrolase/β-glucuronidase | 3/36 |
| **CNBG_5038** | Hypothetical | Kre9/Knh1 family | 11/33 |
| **CNBG_5182** | Glyoxal oxidase | Galactose/glyoxal oxidase | 42/32 |
| **CNBG_5209** | Hypothetical | None | NSSF |
| **CNBG_5294** | Hypothetical | None | 21/33 |
| **CNBG_5804** | Endo-1,3(4)-β-glucanase | Glucanase/glycoside hydrolase | 12/43 |
| **CNBG_6180** | Hypothetical | Kre9/Knh1 family | 4/40 |
| **CNBG_0181** | Hypothetical | None | 5/38 |
| **CNBG_0228** | Glucan-1,3- β-glucosidase | Glycoside hydrolase | 10/50 |
| **CNBG_0269** | Hypothetical | None | 8/45 |
| **CNBG_0806** | Chitin deacetylase (*CDA3*)  MP84 ^(1, 2)^ | Glycoside hydrolase/deacetylase | 9/38 |
| **CNBG_1576** | Hypothetical | None | NSSF |
| **CNBG_1672** | Endopeptidase | Aspartic peptidase A1 | 16/36 |
| **CNBG_2366** | Hypothetical | Kre9/Knh1 family | 4/37 |
| **CNBG_5735** | Hypothetical | None | 10/25 |
| **CNBG_9064** | Chitin deacetylase 2 (*CDA2*)  MP98 ^(2, 3)^ | Glycoside hydrolase/deacetylase | 16/50 |
| **CNBG_2946** | Nuclear protein | Phosphatidylethanolamide-binding protein | 27/34 |
| **CNBG_9160**^II^ | Hypothetical | None | 8/32 |
| **CNBG_0840** | Hypothetical | None | 15/69 |
| **CNBG_2759** | Glycosyl hydrolase | Class I glutamine amido-transferase-like/ThuA-like | 27/34 |
| **CNBG_5802** | Hypothetical | ConA-like lectin/Glucanase/Glycoside hydrolase family 16 | 3/47 |
| **CNBG_5332** | α-amylase | Glycoside hydrolase | 38/23 |
| **CNBG_5392** | Hypothetical | None | NSSF |

**^A^** FungiDB codes

**^B^** InterProscan

**^C^** Sequence coverage (%) **/** sequence identity (%) in relation to *CNBG_4278* sequence

**^D^** No significant similarity found (NSSF)

^I^ The ortholog in *C. neoformans* (CNAG_06000) does not have GPI anchor.

^II^ The ortholog in *C. neoformans* (CNAG_05915) does not have GPI anchor.
