# Supplementary material for: A Predicted Mannoprotein Participates in Cryptococcus gattii Capsular Structure
Source: mSphere. 2018 Apr 25;3(2):e00023-18. doi: 10.1128/mSphere.00023-18 (PMC5917426; doi:10.1128/mSphere.00023-18)
Supplement: TABLE S2 [file sph002182524st2.docx]

**Table S2. *C. neoformans* predicted mannoproteins**

| *C. neoformans* predicted mannoproteins **^A^** | Annotation | Domain prediction **^B^** | Conservation (Coverage/Identity) **^A^** |
| --- | --- | --- | --- |
| **CNAG_05595** | Hypothetical | Kelch | CNBG_4278 |
| **CNAG_00261** | Hypothetical | None | CNBG_0310 |
| **CNAG_00373** | Glucan-1,3- β-glucosidase | Glycoside hydrolase | CNBG_0228 |
| **CNAG_00407** | Glyoxal oxidase | Galactose/glyoxal oxidase | CNBG_1499 |
| **CNAG_00776**^I^ | MP88 ^(4)^ | None | CNBG_1155 |
| **CNAG_01239** | Chitin deacetylase (*CDA3*) MP84 ^(1, 5)^ | Glycoside hydrolase/deacetylase | CNBG_0806 |
| **CNAG_01272** | Hypothetical | None | CNBG_0840 |
| **CNAG_01986**^II^ | Hypothetical | None | CNBG_2966 |
| **CNAG_02030** | Glyoxal oxidase | Galactose/glyoxal oxidase | CNBG_5182 |
| **CNAG_02060** | Hypothetical | None | CNBG_5209 |
| **CNAG_02146** | Hypothetical | Fas1 domain | CNBG_5294 |
| **CNAG_02189** | α-amylase | Glycoside hydrolase | CNBG_5332 |
| **CNAG_02775** | Hypothetical | None | - |
| **CNAG_03223** | Hypothetical | Kre9/Knh1 family | CNBG_2366 |
| **CNAG_03857** | Hypothetical | None | CNBG_1575 |
| **CNAG_03858** | Hypothetical | None | CNBG_1576 |
| **CNAG_04291** | Glycosyl hydrolase | Class I glutamine amido-transferase-like/ThuA-like | CNBG_2759 |
| **CNAG_04357** | Hypothetical | Kre9/Knh1 family | CNBG_2705 |
| **CNAG_04635** | Endopeptidase | Aspartic peptidase A1 | CNBG_1017 |
| **CNAG_04874** | Hypothetical | ConA-like lectin/Glucanase/Glycoside hydrolase family 16 | CNBG_5802 |
| **CNAG_05156** | Hypothetical | CFEM domain | CNBG_4538 |
| **CNAG_05424** | Hypothetical | None | CNBG_4430 |
| **CNAG_05458** | Endo-1,3(4)-β-glucanase | ConA-like lectin/Glucanase/Glycoside hydrolase family 16 | CNBG_4403 |
| **CNAG_05799**^III^ | Chitin deacetylase (*CDA1*)^(2)^ | Glycoside hydrolase/deacetylase | CNBG_1745 |
| **CNAG_06422** | Hypothetical | Kre9/Knh1 family | CNBG_5038 |
| **CNAG_06501**^IV^ | 1,3-β-glucanosyltransferase | Glucanosyltransferase/  glycoside hydrolase/X8-like | CNBG_4970 |
| **CNAG_06795** | Hypothetical | Kre9/Knh1 family | CNBG_6180 |
| **CNAG_07856** | Hypothetical | Quinoprotein alcohol dehydrogenase-like | CNBG_0972 |
| **CNAG_00301** | Hypothetical | None | CNBG_0269 |
| **CNAG_01230** | Chitin deacetylase 2 (*CDA2*)  MP98^(2, 3)^ | Glycoside hydrolase/deacetylase | CNBG_9064 |
| **CNAG_02759** | Hypothetical | None | CNBG_3416 |
| **CNAG_03204** | Hypothetical | None | CNBG_2381 |
| **CNAG_03782** | Hypothetical | None | CNBG_0181 |
| **CNAG_04091** | Hypothetical | None | CNBG_5392 |
| **CNAG_04506** | Hypothetical | None | - |
| **CNAG_04876** | Endo-1,3(4)-β-glucanase | ConA-like lectin/Glucanase/Glycoside hydrolase family 16 | CNBG_5804 |
| **CNAG_05278** | Hypothetical | None | - |
| **CNAG_05312** | Hypothetical | None | CNBG_5735 |
| **CNAG_06396** | Hypothetical | None | - |
| **CNAG_02008** | Nuclear protein | Phosphatidylethanolamide-binding protein | CNBG_2946 |
| **CNAG_07784** | Hypothetical | None | - |
| **CNAG_06104** | Hypothetical | Glycoside hydrolase/β-glucuronidase | CNBG_4814 |
| **CNAG_05872** | Endopeptidase | Aspartic peptidase A1 | CNBG_1672 |

**^A^** FungiDB codes

**^B^** InterProscan

^I^ The ortholog in *C. gattii* (CNBG_1155) does not have signal peptide

^II^ The ortholog in *C. gattii* (CNBG_2966) does not have both signal peptide and GPI anchor

^III^ The ortholog in *C. gattii* (CNBG_1745) does not have signal peptide

^IV^ The ortholog in *C. gattii* (CNBG_4970) does not have GPI anchor
