# Supplementary material for: A Predicted Mannoprotein Participates in Cryptococcus gattii Capsular Structure
Source: mSphere. 2018 Apr 25;3(2):e00023-18. doi: 10.1128/mSphere.00023-18 (PMC5917426; doi:10.1128/mSphere.00023-18)
Supplement: TABLE S3 [file sph002182524st3.docx]

**Table S3: Primers used in the present work**

| Primer | Sequence (5’ - 3’) |
| --- | --- |
| DEL5 F | AAAATAGGGATAACAGGGTAATACTCGGCGTTAACGGCATGG |
| DEL5 R | GGGGACAAGTTTGTACAAAAAAGCAGGCTATCTGTCGGGACCAATGACGTA |
| DEL3 F | GGGGACCACTTTGTACAAGAAAGCTGGGTATACGTCTCAAGACAAAGCTG |
| DEL3 R | AAAAATTACCCTGTTATCCCTATGAGAAGCCTTTTAGAGCC |
| COMP F | AGAAACTCACGTGGAAGACC |
| COMP R | CTGACTGAAATGATCGGTAG |
| RT1 F | GATTGGGGACAACAACACCT |
| RT1 R | GCTGCTCAAGTCGTCGTCGT |
| RT2 F | CCGCGAACAATGTTCCTTACT |
| RT2 R | TGTCTTGAGACGTAGGAGCG |
| RT3 F | ACGACTTGAGCAGCATCTAC |
| RT3 R | GGGTGAAGAATACATTGGTG |
| RT4 F | TCAAGTGACACCCTCAGATG |
| RT4 R | CACCACGAAAATGTAAGCAG |
| pET23 F | AAAAAGGATCCTATGCGTCCAGTTTGACTCG |
| pET23 R | AAAAAAAGCTTAGAATTGGTACTAGCAGAAGCG |
| ACT F | CGGTATCGTCACAAACTGG |
| ACT R | GGAGCCTCGGTAAGAAGAAC |
